# A Mo-doped carbon dot nanozyme for enhanced phototherapy *in vitro*

**DOI:** 10.1039/d5na00028a

**Published:** 2025-02-11

**Authors:** Wenlong Wang, Xuan Sheng, Yihan Wang, Mingjun Yu, Yue Shen, Youfu Xia, Tiao Li, Shuai Cao, Mengjuan Zhang, Wenjian Wang, Yongjian Yang

**Affiliations:** a School of Traditional Chinese Medicine, Bozhou University Anhui 236800 P. R. China 1422905237@qq.com wwj7659996@126.com

## Abstract

Cancer is a leading cause of death globally, and traditional treatment methods often come with non-negligible toxic side effects in its treatment, threatening patients' quality of life. Thus, developing novel, efficient, low-toxicity cancer treatment strategies is crucial. Nanozymes, as a class of powerful nanomaterials, can subtly mimic the catalytic activity of natural enzymes, making them a formidable alternative. Hypoxic molybdenum oxide (MoO_3−*x*_), as a typical nanozyme material, possesses unique physical and chemical properties, showing great potential in fields such as cancer treatment. In this study, a simple and rapid one-pot hydrothermal synthesis method was ingeniously employed, innovatively combining molybdenum, which has high biosafety, with safflower, which exhibits anticancer pharmacological activity, to successfully prepare hypoxic molybdenum oxide (MoO_3−*x*_)-doped safflower carbon dots (H-Mo-CDs). H-Mo-CDs exhibit exceptional catalase (CAT)-like, peroxidase (POD)-like, and superoxide dismutase (SOD)-like catalytic activities and superior photothermal conversion efficiency and photostability. *In vitro* cellular experiments have verified their multiple therapeutic potentials in photothermal therapy (PTT), chemodynamic therapy (CDT), and photodynamic therapy (PDT), providing novel ideas and means for precise cancer treatment. This study not only paves an efficient and feasible path for the development of Mo-based nanomaterials as “smart” nanozymes but also injects new vitality and possibilities into the types and applications of nanozymes in cancer treatment.

## Abstract 2

Cancer is a leading cause of death globally, and traditional treatment methods often come with non-negligible toxic side effects in its treatment, threatening patients' quality of life.

## Graphical abstract

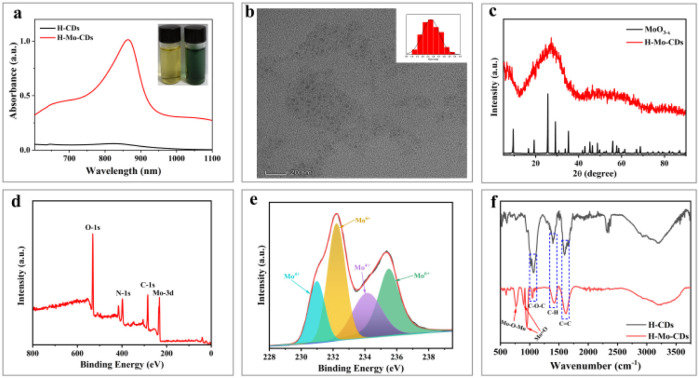

## Introduction

Cancer, a highly aggressive disease, continues to be a leading cause of death worldwide. To date, humanity's fight against this adversary has primarily relied on a multifaceted array of treatment modalities,^1–4^ including surgical intervention, radiotherapy (RT), chemotherapy, targeted therapy, photodynamic therapy (PDT), and photothermal therapy (PTT). These treatments collectively form a formidable barrier against tumor growth and dissemination. Regrettably, however, despite their notable therapeutic efficacy, these modalities are inevitably accompanied by toxic and adverse side effects, adding considerable risks and complexities to the treatment process. Consequently, the exploration and development of newer, more efficient, and less toxic diagnostic and therapeutic methods for cancer have emerged as pressing research priorities in the current medical landscape.

Nanozymes,^5–8^ a fascinating class of functional nanomaterials, distinguish themselves by their ability to mimic the catalytic activity of natural enzymes. Since the pioneering discovery by Yan *et al.* in 2007, which revealed the enzyme-like activity of Fe_3_O_4_ nanoparticles,^9^ nanozymes have swiftly emerged as potent alternatives to natural enzymes due to their high catalytic activity, low cost, mild reaction conditions, exceptional stability, and ease of large-scale production.^10–14^ In recent years, with the deep integration of nanomedicine and nanocatalysis, nanozyme-based therapeutic strategies have garnered widespread attention from researchers worldwide. These strategies are particularly noteworthy for their ability to precisely trigger enzymatic reactions in the tumor microenvironment, achieving efficient treatment with minimal side effects, while demonstrating remarkable substrate specificity.^15^ To date, materials exhibiting nanozyme activity have been primarily classified into oxidoreductases and hydrolases. Among them, oxidoreductases (such as peroxidase (POD), catalase (CAT), oxidase (OXD), superoxide dismutase (SOD), and nitrate reductase) are the most widely utilized types of nanozymes.^5,15–18^ Within the realm of nanozyme materials, transition metal oxides have become a research hotspot due to their early demonstration of peroxidase activity.^19–21^ These materials possess empty d or f orbitals, which facilitate the formation of coordination bonds with substrate molecules, reducing the energy barrier of reaction transition states, effectively accelerating chemical reactions and exhibiting exceptional catalytic therapeutic effects, thereby possessing immense value in catalytic therapy.

Hypoxic molybdenum oxide (MoO_3−*x*_), as a typical transition metal oxide nanomaterial,^22^ possesses unique physical and chemical properties such as low cost, mild synthesis methods, excellent photothermal performance, low toxicity, and local surface plasmon resonance (LSPR),^23^ making it widely applicable in fields such as catalysis,^24^ sensing,^25^ and energy storage.^26^ Based on the degree of oxygen vacancy defects, molybdenum oxides can be classified into stoichiometric MoO_3_, redox MoO_3−*x*_ (2 < *x* < 3), and reduced MoO_2_, with the valence state of molybdenum gradually shifting from +6 to +4 and the color changing from yellow to blue and finally to black.^27^ Furthermore, due to the oxygen defects in molybdenum oxide (MoO_3−*x*_), it can effectively absorb near-infrared laser light and exhibit the LSPR effect, thereby converting laser energy efficiently into heat to ablate cancer cells.^28–34^ This property has successfully broadened the application scope of molybdenum oxide, endowing it with tremendous potential in fields such as drug delivery, biosensing, cancer therapy, and fluorescence imaging. However, MoO_3−*x*_ alone is prone to aggregation and has poor biocompatibility, limiting its application in the biomedical field. Typically, grafting or modifying the oxide surface is considered a common approach to overcome these shortcomings.^35,36^

Chinese herbal medicines, as a vital component of Chinese medicine, have become indispensable resources for human survival due to their unique pharmacological activities and therapeutic effects. Herbal carbon dots (H-CDs) are carbon dots prepared by combining the medicinal components of natural Chinese herbal medicines with the optical properties of traditional carbon dots.^37^ H-CDs not only possess the general characteristics of carbon dots, such as nano-size effects, photoluminescence, ease of surface functionalization, good water solubility, and excellent biocompatibility but also effectively retain the effective bioactive components of Chinese herbal medicines, exhibiting potential efficacy in areas such as radical scavenging, antioxidant activity, antibacterial activity, anti-inflammatory activity, and antitumor activity.^38–41^ Furthermore, research has found that these herbal-derived carbon dots can improve certain properties of the material after synthesis, such as solubility, retain some characteristics of the raw material, or gain new biological activities. Therefore, selecting suitable Chinese herbal medicines as carbon sources for synthesizing carbon dots with fluorescence stability and pharmacological activity provides guidance for exploring the intrinsic biological activities, material composition, and physicochemical properties of herbal-derived carbon dots. Safflower, a traditional Chinese herbal medicine, possesses the effects of promoting blood circulation to remove blood stasis and alleviating pain.^42,43^ It is often used in compound prescriptions in the clinical treatment of various malignant tumors.^44^ In particular, it has achieved good results in the clinical treatment of breast cancer, cervical cancer, gastric cancer, liver cancer, and other conditions characterized by blood stasis congestion and obstruction, as well as obvious pain. The main antitumor components are flavonoids, including safflower yellow, hydroxysafflor yellow A, and safflower polysaccharides, which exhibit good antitumor effects on cancers such as breast cancer, cervical cancer, and gastric cancer^44–46^ (inhibiting tumor cell proliferation, inducing tumor cell apoptosis, inhibiting neovascularization within tumors, *etc.*).

**Scheme 1:**
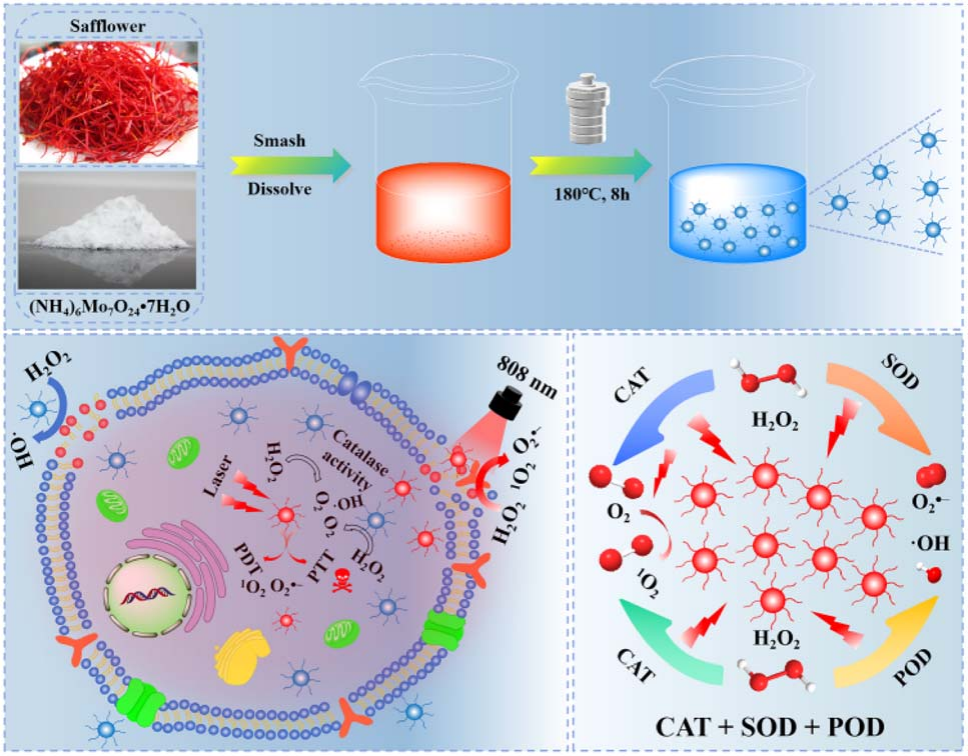
Schematic diagram of the preparation of H-Mo-CDs and their enzymatic activity for enhanced phototherapy of tumor cells.

Herein, we successfully synthesized smaller-sized hypoxic molybdenum oxide (MoO_3−*x*_)-doped safflower carbon dots (H-Mo-CDs) through a facile one-pot hydrothermal synthesis route by combining the highly biocompatible transition metal molybdenum (Mo) with safflower, which possesses notable anticancer activity. Compared to undoped safflower carbon dots, the molybdenum oxide (MoO_3−*x*_) in H-Mo-CDs exhibits superior peroxidase (POD)-like and catalase (CAT)-like catalytic activities, effectively catalyzing the conversion of H_2_O_2_ into highly reactive ^1^O_2_, ˙OH, and O_2_ radicals. Under near-infrared (NIR, 808 nm) light irradiation, H-Mo-CDs not only demonstrate excellent photostability but also possess outstanding photothermal conversion efficiency. *In vitro* cellular experiments further validate the dual therapeutic potential of H-Mo-CDs: they not only induce significant photothermal therapeutic effects under NIR light irradiation but also catalyze the conversion of excess H_2_O_2_ within cancer cells into highly cytotoxic ^1^O_2_, ˙OH, and O_2_ radicals, thereby activating chemodynamic therapy (CDT) to synergistically induce cancer cell apoptosis. In summary, H-Mo-CDs, as a dual therapeutic platform integrating CDT and phototherapy, exhibit significant academic value and application potential in the field of cancer photodiagnosis and treatment (Scheme 1).

## Results and discussion

### Synthesis and structural characterization of H-Mo CDs

**Fig. 1:**
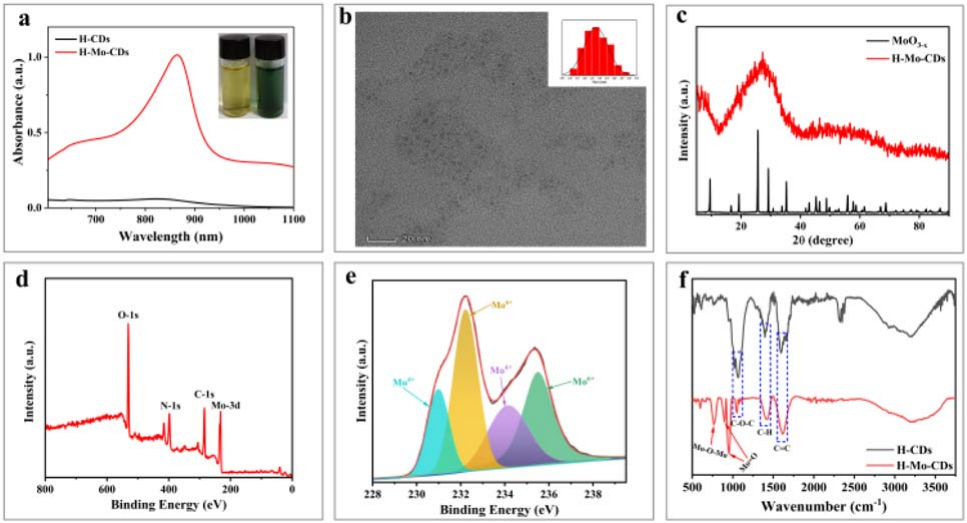
The UV-Vis-NIR absorption spectra of H-Mo-CDs (a); TEM images of H-Mo-CDs (b); XRD patterns of CH-Mo-CDs (c); XPS survey spectrum of H-Mo-CDs (d); the XPS fine spectrum of Mo 3d (e); FT-IR spectra (f).

H-Mo CDs were synthesized using a straightforward one-step hydrothermal method involving safflower powder and (NH_4_)_6_Mo_7_O_24_·7H_2_O (Scheme 1). Post-synthesis, the UV-visible absorption spectrum of the synthesized H-Mo CDs was analyzed to investigate their absorption behavior in water. H-Mo CDs exhibited strong narrow peak absorption around 864 nm. Visual inspection of the absorption changes (inset of Fig. 1a) also revealed that the color of the safflower carbon dots (H-CDs) was yellow, whereas the color of H-Mo-CDs was green, further indicating the formation of H-Mo-CDs. Therefore, doping with (NH_4_)_6_Mo_7_O_24_·7H_2_O not only addressed the water solubility of Mo-based materials but also significantly enhanced their absorption in the near-infrared region. Transmission electron microscopy (TEM) images of H-Mo-CDs demonstrated good dispersion and a relatively narrow size distribution, with quantum dots composed of particles ranging from 2.0 to 4.0 nm, as shown in Fig. 1b. Studies have shown that small-sized nanoparticles can evade capture by the reticuloendothelial system, thereby accumulating at tumor sites for optimal therapy.^47^ Additionally, the prepared nanoparticles exhibited indistinct lattice stripes, indicating an amorphous morphology. X-ray diffraction (XRD) analysis of the phase composition of H-Mo-CDs (Fig. 1c) revealed no characteristic peaks of MoO_3−*x*_ in the XRD curve of H-Mo-CDs. Based on previous reports on XRD results of MoO_3−*x*_, the absence of characteristic peaks may be attributed to the small size of the particles and the significant presence of inorganic carbon materials,^30,48^ which may have passivated or even caused the disappearance of the XRD characteristic peaks of MoO_3−*x*_. The XPS spectrum of H-Mo-CDs, shown in Fig. 1d, further verified the presence of Mo. Referring to binding energy tables and related research literature,^27–33,48^ the valence states of Mo in the Mo 3d spectrum were analyzed and assigned, as shown in Fig. 1e. The binding energies at 231.0 eV and 234.3 eV belonged to Mo(v) 3d_5/2_ and Mo(v) 3d_3/2_, respectively, while the binding energies at 233.1 eV and 235.8 eV corresponded to the higher valence states of Mo(v) 3d_5/2_ and Mo(vi) 3d_3/2_, respectively. Thus, the XPS spectrum indicated the coexistence of Mo(v) and Mo(vi) in H-Mo-CDs, providing further theoretical support for their application in enzymatic activity. Fourier Transform Infrared (FT-IR) spectroscopy has further substantiated the successful modification of MoO_3−*x*_ within H-Mo-CDs (Fig. 1f). The peak at 1600 cm^−1^ corresponds to the stretching vibration of C=C bonds; the absorption peak near 1406 cm^−1^ arises from the in-plane bending vibration of C–H bonds; and the peak at 1050 cm^−1^ signifies the stretching vibration of C–O–C bonds. Notably, the peak at 760 cm^−1^ is attributed to the stretching vibration of Mo–O–Mo bonds, and the peaks at 902 cm^−1^ and 947 cm^−1^ represent the characteristic stretching vibrations of Mo=O bonds.^28,29,49,50^ These test results are largely consistent with previous research findings. They not only affirm the significant interaction between H-CDs and the MoO_3−*x*_ surface but also highlight the importance of such interaction for optimizing the properties and potential applications of the composite material.

### Photothermal performance and photothermal stability testing

**Fig. 2:**
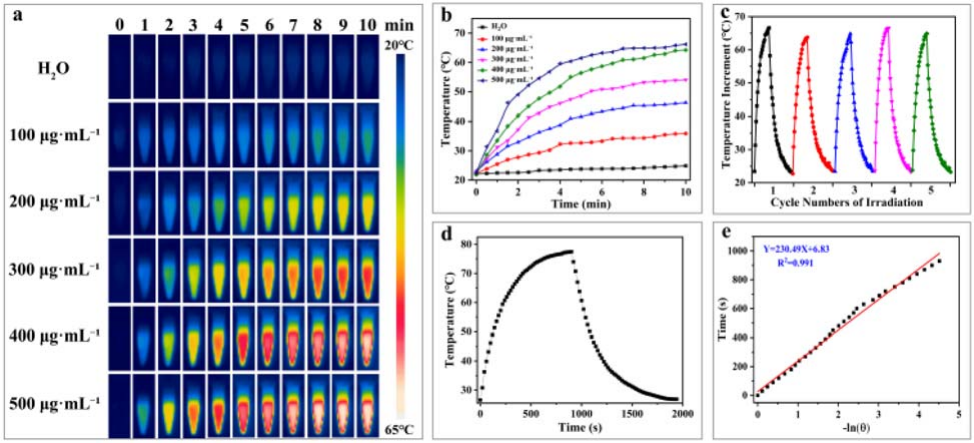
The corresponding time-dependent photothermal curves of samples (a) and photothermal images of H-Mo-CDs and water upon exposure to an 808 nm laser for different time periods (b); heating/cooling curve of H-Mo-CDs over five cycles (c); a single heating/cooling curve (d) and the corresponding plot of cooling time *vs.* the negative natural logarithm of driving force temperature (e). (808 nm, 0.8 W cm^−2^).

In the biomedical field, the photothermal stability of photothermal nanomaterials is crucial for successful application, directly impacting their therapeutic efficacy in physiological environments. First, the photothermal heating performance of H-Mo-CDs was evaluated. Upon irradiation with an 808 nm laser (0.8 W cm^−2^) for 10 minutes, a significant and concentration-dependent temperature increase was observed, with a temperature change of up to 43.7 °C, far exceeding the 2.8 °C increase observed in the control group (Fig. 2a and b). This confirmed that H-Mo-CDs can effectively absorb 808 nm laser light and convert it into thermal energy, suggesting potential photothermal killing effects on tumor cells. Furthermore, the photothermal stability of H-Mo-CDs was investigated. During five cycles of laser irradiation and cooling (Fig. 2c), the H-Mo-CDs solution rapidly increased in temperature to approximately 44 °C with minimal temperature fluctuations and no precipitation formed, indicating good photothermal stability and favoring repeated applications in photothermal therapy. Additionally, the photothermal heating and cooling curves of an H-Mo-CDs aqueous solution at a concentration of 500 μg mL^−1^ under the same laser conditions were measured (Fig. 2d). The temperature increased from 22.6 °C to 77.5 °C, with a photothermal conversion efficiency of up to 46.45% (Fig. 2e). These results further confirm the great potential of H-Mo-CDs in the biomedical field, particularly for photothermal therapy.

### Peroxidase-like and catalase-like activities

**Fig. 3:**
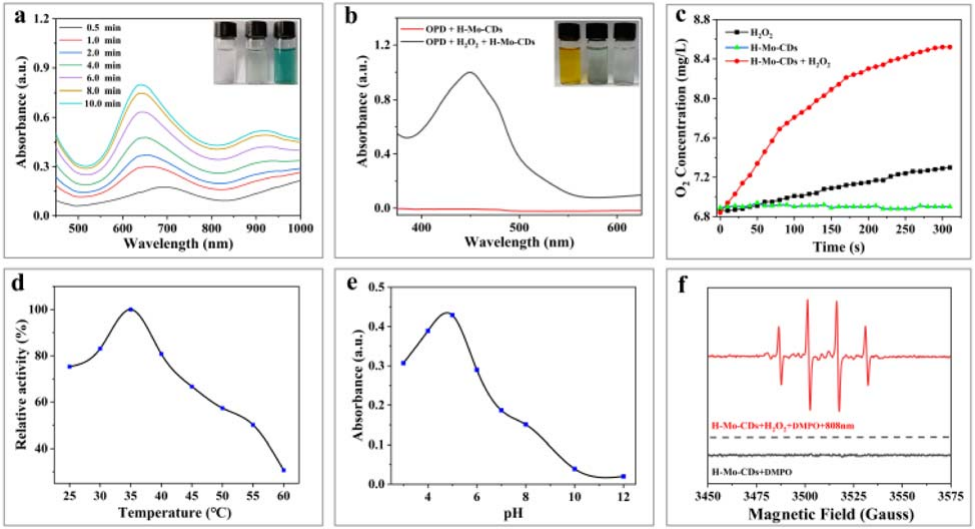
Time-dependent absorbance changes of TMB after different treatments (a); the UV-Vis absorbance changes of OPD (b); time-dependent O_2_ production of H_2_O_2_, H-Mo-CDs, and H-Mo-CDs + H_2_O_2_ in water (c); effect of temperature on peroxidase activity of H-Mo-CDs (d); pH-dependent absorbance changes of H-Mo-CDs + H_2_O_2_ (e); ESR spectra of DMPO after different treatments (f).

In the presence of H_2_O_2_, 3,3′,5,5′-tetramethylbenzidine (TMB), a typical chromogenic substrate for peroxidase oxidation was selected to study the peroxidase-like activity of H-Mo-CDs. As shown in Fig. 3a, H-Mo-CDs exhibited excellent peroxidase-like activity. Additionally, *o*-phenylenediamine (OPD), another typical peroxidase chromogenic substrate, was also used to verify the peroxidase-like activity of H-Mo-CDs (Fig. 3b), further confirming their significant peroxidase-like activity. Electron Spin Resonance (ESR) measurements were further employed to demonstrate the production of ˙OH by H-Mo-CDs in the presence of H_2_O_2_ catalysis (Fig. 3f). Beyond peroxidase-like activity testing, we also confirmed that H-Mo-CDs possess the ability to decompose H_2_O_2_ to generate O_2_ (Fig. 3c). When H-Mo-CDs were added to an H_2_O_2_ solution, O_2_ was rapidly produced, while no O_2_ was generated in the absence of H_2_O_2_, indicating that H-Mo-CDs also exhibit catalase-like activity. Since the catalytic activity of enzymes is related to pH and temperature, to better demonstrate their enzymatic activity, we analyzed the enzymatic activity of H-Mo-CDs at different pH levels and temperatures (Fig. 3d and e). Notably, it was found that H-Mo-CDs maintained good catalytic activity under physiological conditions.

**Fig. 4:**
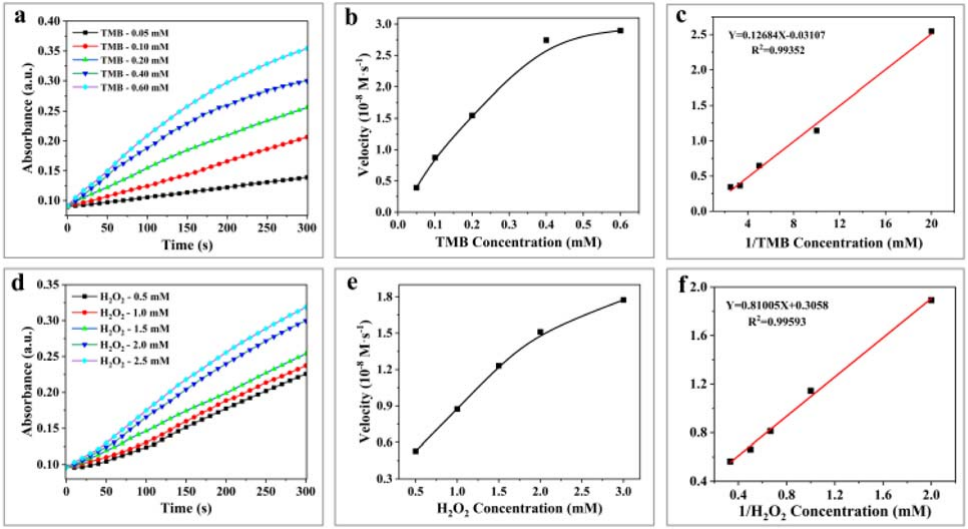
Steady-state kinetic assay of H-Mo-CDs. Time-dependent absorbance changes at 652 nm of TMB reaction solutions catalyzed by H-Mo-CDs in the presence of different concentrations of TMB (a) or H_2_O_2_ (d). The velocity (*v*) of the reaction changes in the presence of different concentrations of TMB (b) or H_2_O_2_ (e). Double reciprocal plots of the activity of H-Mo-CDs in the presence of different concentrations of TMB (c) or H_2_O_2_ (f).

By establishing a Michaelis–Menten model, we further quantified the peroxidase-like activity of H-Mo-CDs using different concentrations of TMB and H_2_O_2_ as variables and obtained typical Michaelis–Menten curves. The kinetic parameters of H-Mo-CDs, including the maximum initial velocity (*V*_max_) and the Michaelis–Menten constant (*K*_m_), were determined. The *K*_m_ value reflects the binding affinity between the enzyme and the substrate, while the *V*_max_ value indicates the turnover number of the enzyme, reflecting its biocatalytic activity. Through linear fitting, the *V*_max_ values of H-Mo-CDs for H_2_O_2_ and TMB were found to be 3.27 × 10^−8^ M s^−1^ and 3.22 × 10^−7^ M s^−1^, respectively, with *K*_m_ values of 2.65 mM and 4.082 mM. These results (Fig. 4) indicate that H-Mo-CDs exhibit kinetic parameters similar to those of typical horseradish peroxidase (HRP) and Fe_3_O_4_ magnetic nanoparticles (MNPs),^9,21^ demonstrating significant nanozyme-like activity. Therefore, H-Mo-CDs can serve as potential candidates for the development of enzyme mimics.

### Photodynamic activity of H-Mo-CDs

**Fig. 5:**
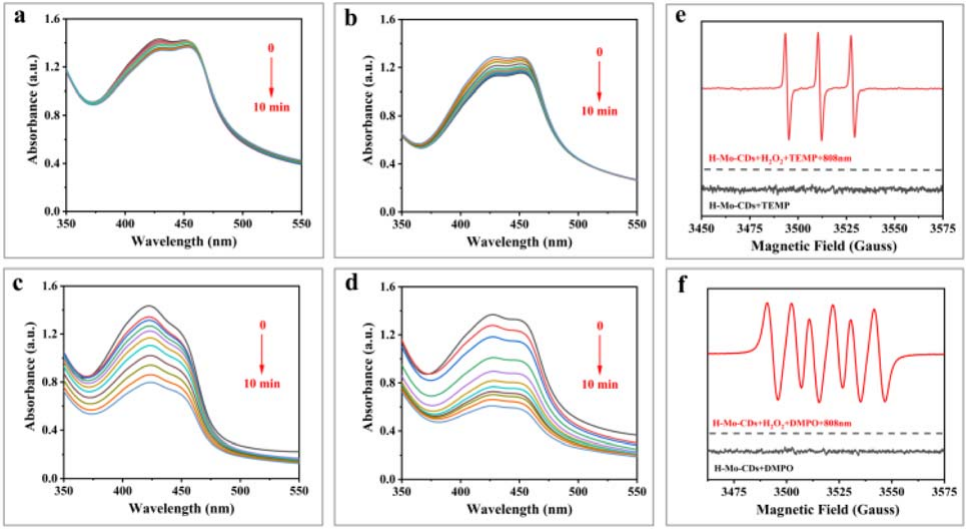
UV-Vis absorption spectra of DPBF after irradiation for different times with an 808 nm laser (a); absorption spectrum changes of DPBF with H-Mo-CDs in the presence of H_2_O_2_ (b); absorption spectrum changes of DPBF under 808 nm laser irradiation with H-Mo-CDs in the absence (c) or presence (d) of H_2_O_2_; electron spin resonance (ESR) spectra of H-Mo-CDs in the presence of TEMP (e) and DMPO (f).

1,3-Diphenylisobenzofuran (DPBF) serves as a chemical scavenger of reactive oxygen species (ROS) and can detect the ability of materials to generate ROS under light illumination. DPBF undergoes a 1,4-cycloaddition reaction upon encountering ROS, resulting in a decrease in its absorbance at 418 nm.^3,21,29^ In this study, a DPBF aqueous solution (pH = 4.5) was used to detect whether H-Mo-CDs generate ROS under light illumination or in the presence of H_2_O_2_. Fig. 5a–d show that the absorbance of DPBF at 418 nm decreased significantly when H-Mo-CDs were combined with 808 nm laser light or H_2_O_2_, and the decrease was more pronounced when both were present. To further identify the types of ROS, electron spin resonance (ESR) technology was employed using 5,5-dimethyl-1-pyrroline *N*-oxide (DMPO) and 2,2,6,6-tetramethylpiperidine (TEMP) as scavengers for ˙OH (or O_2_˙^−^) and ^1^O_2_, respectively. The results in Fig. 5e and f indicate that the ESR sextet signal of DMPO–OOH was detected when H_2_O_2_ and H-Mo-CDs coexisted, attributed to the production of O_2_˙^−^ by H-Mo-CDs catalyzing H_2_O_2_. Simultaneously, a triplet signal was observed, attributed to the production of ^1^O_2_ by H-Mo-CDs catalyzing H_2_O_2_. Combining the above enzyme-like activity results, it is inferred that H-Mo-CDs promote ROS generation in the H_2_O_2_ + 808 nm laser group, which originates from both the peroxidase-like activity of H-Mo-CDs producing ˙OH and their catalase-like activity catalyzing the production of O_2_˙^−^ to induce enhanced ROS generation.

### Assessment of cytotoxicity and *in vitro* photothermal effect of H-Mo-CDs

**Fig. 6:**
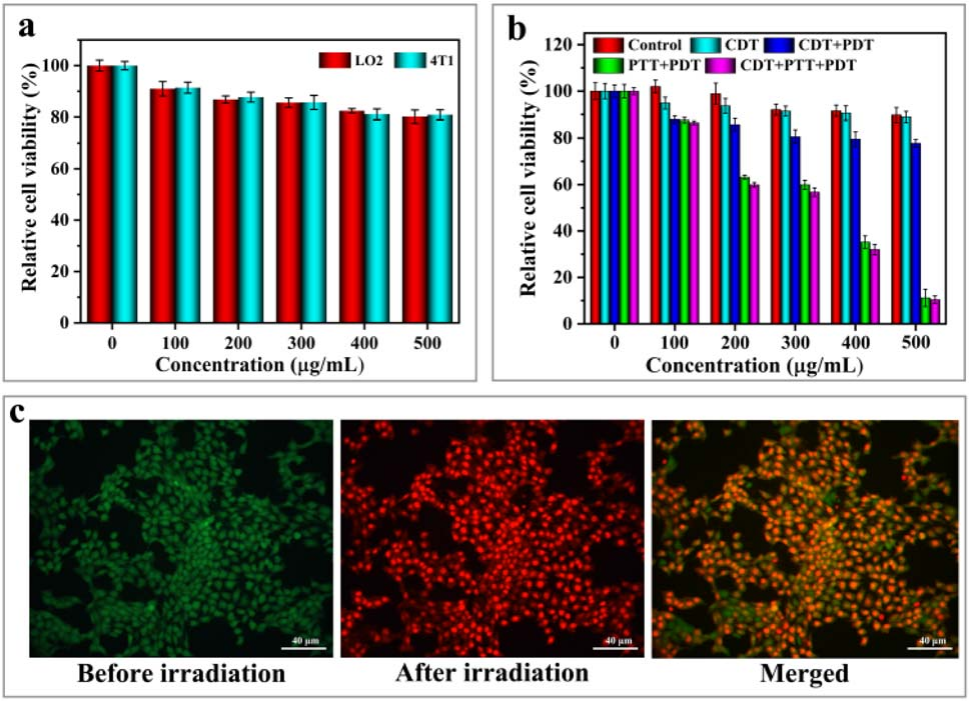
Relative viabilities of LO2 and 4T1 cells incubated with different concentrations of H-Mo-CDs: non-irritated light (a); the relative viability of 4T1 cells and their synergistic effects on different concentrations of H-Mo-CDs were induced in the CDT group (plus H_2_O_2_), CDT + PDT group (plus H_2_O_2_ and 808 nm laser irradiation under ice), PTT + PDT group (808 nm laser irradiation only) and CDT + PTT + PDT group (plus H_2_O_2_ and 808 nm laser irradiation) (b); confocal images of 4T1 cells co-stained with calcein AM and PI before and after irradiation with an 808 nm laser (c). Scale bar: 40 μm.

The low cytotoxicity of phototherapeutic agents is a crucial factor in determining their feasibility for implementation in the biomedical field. In this experiment, 3-(4,5-dimethylthiazol-2-yl)-2,5-diphenyltetrazolium bromide (MTT) was used to further assess the *in vitro* cytotoxic effects of H-Mo-CDs. H-Mo-CDs were co-incubated with LO2 (human liver cells) and 4T1 (murine breast cancer cells) to test for *in vitro* cytotoxicity. During this process, H-Mo-CDs were set at concentrations of 100 μg mL^−1^, 200 μg mL^−1^, 300 μg mL^−1^, 400 μg mL^−1^, and 500 μg mL^−1^ and co-cultured with both cell types for 24 hours before conducting standard MTT assays to determine relative cell viability. As shown in Fig. 6a, even at a maximum concentration of 500 μg mL^−1^, the cell viability of both groups was above 80%, indicating that H-Mo-CDs exhibit low cytotoxicity to a certain extent. It is well-known that abnormal metabolism in cancer cells within solid tumors constitutively produces H_2_O_2_.^51,52^ Therefore, to mimic the H_2_O_2_-rich tumor microenvironment, the *in vitro* anticancer efficiency of H-Mo-CDs was further evaluated in the presence of externally added 1 mM H_2_O_2_. Next, H-Mo-CDs were incubated with 4T1 cells, and 808 nm laser irradiation and 1 mM H_2_O_2_ were applied to investigate the therapeutic efficacy of the *in vitro* phototherapeutic agent. The results in Fig. 6b show that, under conditions mimicking the H_2_O_2_-rich tumor microenvironment, the cell viability of 4T1 cells decreased slightly (Fig. 6b CDT group). This demonstrates the feasibility of H-Mo-CDs catalyzing H_2_O_2_ to produce ˙OH for killing cancer cells. With the combination of 808 nm laser irradiation and H_2_O_2_ (Fig. 6b CDT + PDT + PTT group), a significant decrease in cell viability was observed. Under the synergistic effect of CDT/PDT/PTT, over 80% of 4T1 cells were killed.

As an auxiliary method to measure therapeutic efficacy, the live/dead cell staining method was employed in this experiment using calcein-AM and propidium iodide (PI) to label live and dead cells, respectively, further validating the therapeutic effect of H-Mo-CDs. 4T1 cells were incubated with H-Mo-CDs and then treated with calcein-AM and PI. As shown in Fig. 6c, after 6 minutes of laser irradiation, 4T1 cells exhibited significant red fluorescence indicative of dead cells, which is in good agreement with the above MTT assay results (Fig. 6b). This further demonstrates that the synergistic therapeutic effect of H-Mo-CDs in the presence of light can efficiently kill cancer cells, suggesting that H-Mo-CDs can be used as potential photothermal agents.

## Conclusions

In summary, we have successfully developed H-Mo-CDs that synergize CDT with PTT and PDT. The molybdenum component in this nanomaterial not only exhibits satisfactory photothermal conversion ability but also catalyzes the production of O_2_ from H_2_O_2_ in tumor tissue to alleviate tumor hypoxia. Simultaneously, it catalyzes the generation of cytotoxic ROS for CDT and PDT. *In vitro* cellular experiments have demonstrated that H-Mo-CDs can effectively inhibit cell proliferation under 808 nm laser irradiation, revealing their potential PTT/PDT/CDT effects in cancer treatment. Overall, this study provides an efficient and feasible approach for developing Mo-based nanomaterials as “smart” nanozymes, enriching the variety of nanozymes in cancer therapy.

## Experimental

### Materials

All chemicals utilized were of analytical grade and required no further purification. Safflower was obtained from the Bozhou Traditional Chinese Medicine Material Market (Bozhou, Anhui). Ammonium molybdate tetrahydrate ((NH_4_)_6_Mo_7_O_24_·7H_2_O), acetic acid (CH_3_COOH), sodium acetate (CH_3_COONa), 3,3′,5,5′-tetramethylbenzidine (TMB), 1,2-phenylenediamine (OPD), and 1,3-diphenylisobenzofuran (DPBF) were purchased from Source Leaf (Shanghai, China). Human normal liver cells (LO2) and mouse breast cancer cells (4T1) were acquired from the Shanghai Institute of Cell Biology, Chinese Academy of Sciences. All solutions were prepared using deionized water.

### Characterization

The morphology of the samples was examined using a Tecnai G2 F20 S-TWIN (200 kV) Transmission Electron Microscope (TEM). X-ray Photoelectron Spectroscopy (XPS) measurements were conducted on a Thermo Scientific Escalab 250Xi spectrometer. The structural properties of the samples were determined using a D Max-3c X-ray powder diffractometer. Fourier transform infrared spectroscopy (FT-IR) spectra were recorded on a PES spectrum infrared spectrophotometer using the KBr pellet technique. UV-Vis-NIR spectra were recorded using a UV-2600 spectrophotometer.

### Synthesis of H-Mo CDs

To prepare the H-Mo CDs, 0.250 g safflower powder and 0.085 g of (NH_4_)_6_Mo_7_O_24_·7H_2_O were dissolved in 25 milliliters of Milli-Q water. The mixture was then sonicated for 5 minutes and stirred at room temperature for 30 minutes. Subsequently, the solution was transferred into a 30 milliliter polytetrafluoroethylene-lined autoclave and heated at 180 °C for 8 hours in a constant-temperature oven. After the reaction, the obtained solution was dialyzed in a dialysis bag with a molecular weight cut-off (MWCO) of 1000 Da for 48 hours in deionized water to purify it. Finally, the product was freeze-dried to yield water-soluble H-Mo CDs.

### *In vitro* photothermal performance testing

To evaluate the photothermal properties, 0.85 milliliters of aqueous solutions containing H-Mo CDs at concentrations of 100, 200, 300, 400, and 500 μg mL^−1^, as well as ultrapure water, were exposed to an 808 nm laser at a power density of 0.8 W cm^−2^ for 10 minutes. An infrared thermal imager was used to record the temperature increase of the samples in real-time. Additionally, 500 μg mL^−1^ H-Mo CDs underwent five cycles of laser irradiation and temperature changes were recorded according to the aforementioned method.

To determine the photothermal conversion efficiency (*η*), an aqueous solution of H-Mo CDs at a concentration of 500 μg mL^−1^ was irradiated with an 808 nm laser until the temperature stabilized. Subsequently, the laser was turned off, and the temperature of the solution was measured every 10 s until it cooled to room temperature. A control experiment (H_2_O) was conducted under identical conditions.

### Determination of peroxidase-like activity and kinetic parameters

Analogous to natural peroxidases, H-Mo CDs exhibit peroxidase-like activity by catalyzing the reaction between the substrate H_2_O_2_ and 3,3′,5,5′-tetramethylbenzidine (TMB) to produce a blue product. To investigate the peroxidase-like activity of H-Mo CDs, a dependency curve of the ultraviolet (UV) absorption of the product oxTMB as a function of time was analyzed in a reaction system containing 2 mL of a buffer solution with 100 μg mL^−1^ H-Mo CDs (acetic acid-sodium acetate buffer, pH = 4.5), 0.125 mM TMB, and 1 mM H_2_O_2_. Kinetic assessments were also conducted in HAc/NaAc buffer solutions containing 100 μg mL^−1^ H-Mo CDs to evaluate the enzymatic catalytic activity at varying concentrations of μg mL^−1^ (0.5, 1.0, 1.5, 2.0, and 2.5 mM) and TMB (50, 100, 200, 400, and 800 μM).

### Catalase-like activity assessment

The catalase-like activity of H-Mo CDs was compared by measuring the production of O_2_ using an oxygen probe (JPBJ-608 Portable Dissolved Oxygen Meter). H-Mo CDs were dispersed in water containing 1 mM H_2_O_2_, and the oxygen content was monitored over time. This was compared to a dispersion without H_2_O_2_ and H-Mo CDs to serve as a control.

### Electron spin resonance (ESR) spectroscopy tests

#### Measurement of hydroxyl radicals (˙OH) and superoxide anion radicals (O_2_˙^−^)

In a centrifuge tube, 15 μL of a 2.0 M aqueous solution of 5,5-dimethyl-1-pyrroline *N*-oxide (DMPO) was added to 100 μL of an H-Mo CD suspension at a concentration of 25 μg mL^−1^. The resulting solution was irradiated with an 808 nm laser at a power density of 0.8 W cm^−2^ for 5 minutes. The mixture was then injected into a glass capillary tube for immediate ESR measurement.

#### Measurement of singlet oxygen (^1^O_2_)

Similarly, in another centrifuge tube, 10 μL of a 0.8 M aqueous solution of 2,2,6,6-tetramethyl-4-piperidine (TEMP) was added to 100 μL of the same H-Mo CD suspension. The solution was irradiated with the 808 nm laser at the same power density for 5 minutes. The mixture was then injected into a glass capillary tube for immediate ESR measurement.

### Cellular experiments

LO2 cells were cultured in DMEM high-glucose medium, while 4T1 cells were cultured in RPMI 1640 low-glucose medium. Both cell lines were incubated in a humidified incubator at 37 °C with 5% CO_2_. LO2 and 4T1 cell suspensions, at a logarithmic growth phase and a density of 1.0 × 10^5^ cells per mL, were seeded into 96 well plates. After the cells adhered to the plate, the medium was discarded, and fresh medium containing various concentrations of H-Mo CDs was added to each well. Following a 24-h incubation period, cell viability was assessed using the MTT assay according to standard protocols.

Additionally, to evaluate the efficacy of *in vitro* chemodynamic therapy (CDT) and phototherapy, 4T1 cells were incubated with different concentrations of H-Mo CDs for 12 hours. Subsequently, the cells were irradiated with an 808 nm laser at a power density of 0.8 W cm^−2^ for 5 minutes. Cell viability was then assessed using the MTT assay to evaluate *in vitro* cytotoxicity.

### Live/dead cell staining

Following the methods described in the literature, cells were fluorescently labeled using a combination of calcein-AM and propidium iodide (PI) fluorescent detection reagents. Laser scanning confocal microscopy was employed to capture images and assess cell morphology, differentiating between live and dead cells based on their fluorescence patterns.

## Data availability

The data supporting this article have been included as part of the Results and discussion.

## Conflicts of interest

There are no conflicts to declare.
